# Supplementary material for: Proteomic Network Analysis of Bronchoalveolar Lavage Fluid in Ex-Smokers to Discover Implicated Protein Targets and Novel Drug Treatments for Chronic Obstructive Pulmonary Disease
Source: Pharmaceuticals (Basel). 2022 May 1;15(5):566. doi: 10.3390/ph15050566 (PMC9147475; doi:10.3390/ph15050566)
Supplement: Supplementary file 1 [file pharmaceuticals-15-00566-s001.zip › 2022_04_05_BALF_supplemental_response.pdf]

Supplementary material.

Supplementary methods

Inclusion/exclusion criteria[1]

For the COPD group, exclusion criteria included a forced expiratory volume at 1 s (FEV_1) of <35%, hypercapnia, and comorbid diseases that would render bronchoscopy unsafe. Inclusion criteria were (1) chronic bronchitis as determined by history and/or emphysema as determined by chest x-ray or computed tomography; (2) 20 pack-years of cigarette smoking but smoking cessation for at least 1 year preceding enrollment; (3) the absence of other lung disease, including asthma and bronchiectasis; (4) chest x-ray findings that were normal or compatible with COPD but no other disease detected; (5) FEV_1:forced vital capacity ratio and FEV_1 both below the lower 95% confidence limit of normal on spirometry; (6) no atopy in the medical history; (7) <15% bronchodilator response with inhaled albuterol on spirometry; and (8) no antibiotic or steroid use for 4 weeks preceding enrollment. Inclusion criteria for the no-COPD group were the same as those for the COPD group, except for the absence of lung disease by clinical evaluation, normal chest x-ray, and normal spirometric results. Healthy nonsmokers met all inclusion criteria for the no-COPD group, except that all had <5 cumulative pack-years of smoking.

Supplementary tables

Table S1: Drugs commonly prescribed for stable COPD. Curated drugs currently indicated for COPD ("MESH:D029424") from the Comparative Toxicogenomics Database[2] and manually curated to include drugs used to treat stable COPD in the United States.

beclomethasone dipropionate
budesonide
arformoterol
roflumilast
aclidinium
fluticasone propionate
dexamethasone acetate
prednisolone tebutate
prednisone acetate
prednisolone acetate
mometasone
fluticasone
methyl prednisone
pirbuterol
ciclesonide
erdosteine
terbutaline
fluticasone furoate
glycopyrronium
indacaterol
ipratropium
levosalbutamol
clarithromycin
olodaterol
salbutamol

salmeterol
revefenacin
theophylline
salbutamol
aminophylline
umeclidinium
vilanterol
prednisone
tiotropium

Table S2: Unique proteins upregulated in BALF (n=95), Differentially expressed proteins with at least 1.5x fold change increase in the BALF proteome in COPD versus control cohort samples.

Uniprot ID	Symbol	Entrez Gene Name	Location	Type(s)	Fold change	Previously described association with COPD	References
P00325	ADH1B	alcohol dehydrogenase 1B (class I), beta polypeptide	Cytoplasm	Lipid metabolism	12.86	NONE	
P46439	GSTM5	glutathione S-transferase mu 5	Cytoplasm	enzyme	11.26	Various GST polymorphisms implicated in lung protection	3-5
O00487	PSMD14	proteasome (prosome, macropain) 26S subunit, non-ATPase, 14	Cytoplasm	peptidase	9.53	NONE	

Q86TZ1	TTC6	tetratricopeptide repeat domain 6	unknown	other	8.68	NONE	
P02743	APCS	amyloid P component, serum	Extracellular Space	pentraxin	8.23	Not APCS but Pentraxin 3 in COPD	6-8
Q9UBR2	CTSZ	cathepsin Z	Cytoplasm	peptidase	7.92	NONE	
Q9NP98	MYOZ1	myozenin 1	Cytoplasm	other	7.08	NONE	
Q13402	MYO7A	myosin VIIA	Cytoplasm	enzyme	6.84	NONE	
Q49MG5	MAP9	microtubule-associated protein 9	unknown	other	6.31	NONE	
Q13642	FHL1	four and a half LIM domains 1	Cytoplasm	other	5.53	NONE	
P02775	PPBP	pro-platelet basic protein (chemokine (C-X-C motif) ligand 7)	Extracellular Space	cytokine	5.47	Neutrophil marker increased in severe stable COPD	9
P21333	FLNA	filamin A, alpha	Cytoplasm	other	5.14	NONE	
Q8NFC6	BOD1L1	biorientation of chromosomes	Extracellular Space	other	4.43	NONE	

		in cell division 1-like 1					
P04220	MUCB	Ig mu heavy chain disease protein	Plasma Membrane		4.36	NONE	
P02656	APOC3	apolipoprotein C-III	Extracellular Space	Lipid metabolism	4.24	NONE	
P42330	AKR1C3	aldo-keto reductase family 1, member C3 (3-alpha hydroxysteroid dehydrogenase, type II)	Cytoplasm	enzyme	4.11	NONE	
P35749	MYH11	myosin, heavy chain 11, smooth muscle	Cytoplasm	other	4.01	Myosin heavy chain variation was noted in COPD but not specific proteins	[10-12]
O00522	KRIT1	KRIT1, ankyrin repeat containing	Plasma Membrane	other	3.89	NONE	
A6NDU8	CE051	UPF0600 protein C5orf51	unknown	other	3.56	NONE	
P04114	APOB	apolipoprotein B (including Ag(x) antigen)	Extracellular Space	transporter	3.556	NONE	

Q5XKE5	KRT79	keratin 79	Extracellular Space	other	3.43	NONE	
Q9NWS1	PARPBP	PARP1 binding protein	Nucleus	other	3.42	NONE	
P12429	ANXA3	Annexin A3			3.34	NONE	
P30838	ALDH3A1	aldehyde dehydrogenase 3 family, member A1	Cytoplasm	Lipid metabolism	3.33	None, but is implicated in cell proliferation	
Q6NUK1	SLC25A24	solute carrier family 25 (mitochondrial carrier; phosphate carrier), member 24	Cytoplasm	other	3.33	NONE	
P00738	HP	haptoglobin	Extracellular Space	peptidase	3.22	Acute phase reactant associated with COPD	[13,14]
Q16787	LAMA3	laminin, alpha 3	Extracellular Space	other	3.2	Haemophilus and Moraxella binds to laminin	

P02675	FGB	fibrinogen beta chain	Extracellular Space	other	3.17	Serum fibrinogen levels in COPD associated with exacerbations	[14-20]
P08670	VIM	vimentin	Cytoplasm	cytoskeleton component	3.12	Epithelial to mesenchymal transition	[21-25]
Q7KZI7	MARK2	MAP/microtubule affinity-regulating kinase 2	Cytoplasm	kinase	3.12	NONE	
Q15847	APM2	Adipose most abundant gene transcript 2 protein			3.01	NONE	
Q16280	CNGA2	cyclic nucleotide gated channel alpha 2	Plasma Membrane	ion channel	3.01	NONE	
O94782	USP1	ubiquitin specific peptidase 1	Cytoplasm	peptidase	2.9	NONE	
Q96C24	SYTL4	synaptotagmin-like 4	Cytoplasm	transporter	2.82	NONE	
P02671	FIBA	Fibrinogen alpha chain			2.78	Serum fibrinogen levels in COPD associated with exacerbations	[14-20]
P02679	FGG	fibrinogen gamma chain	Extracellular Space	other	2.75	Serum fibrinogen levels in COPD associated with exacerbations	[14-20]

P53004	BLVRA	biliverdin reductase A	Cytoplasm	enzyme	2.66	NONE	
Q2TVT3	KGFLP2	keratinocyte growth factor-like protein 2	unknown	other	2.62	NONE	
P23284	PPIB	peptidylprolyl isomerase B (cyclophilin B)	Cytoplasm	enzyme	2.55	NONE	
P17931	LGALS3	lectin, galactoside-binding, soluble, 3	Extracellular Space	other	2.52	Increased Gal-3 in small airways	26,27
P13645	K1C10	Keratin type I cytoskeletal 10			2.47	NONE	
Q9NX58	LYAR	Ly1 antibody reactive homolog (mouse)	Plasma Membrane	other	2.37	NONE	
O95497	VNN1	vanin 1	Plasma Membrane	Enzyme/lipid metabolism	2.34	NONE	
P16152	CBR1	carbonyl reductase 1	Cytoplasm	enzyme	2.34	NONE	
P23771	GATA3	GATA binding protein 3	Nucleus	transcription regulator	2.33	NONE	
P01023	A2M	alpha-2-macroglobulin	Extracellular Space	transporter	2.31	A protease inhibitor that has increased serum levels found in	13,28-31

						patients with alpha-1 antitrypsin deficiency	
Q15149	PLEC	plectin	Cytoplasm	other	2.31	NONE	
P62328	TYB6	Thymosin beta_4			2.28	NONE	
Q6UXR4	SERPINA13	serpin peptidase inhibitor, clade A (alpha-1 antiproteinase, antitrypsin), member 13 (pseudogene)	Extracellular Space	other	2.26	A serpin peptidase inhibitor that is in the same family of peptidase inhibitor as alpha-1 antitrypsin (a serpin peptidase inhibitor, clade A, member 1) implicated in protease-antiprotease homeostasis	[32,33]
Q14019	COTL1	coactosin-like 1	Cytoplasm	other	2.25	NONE	
P03950	ANGI	Angiogenin			2.24	Increased in induced sputum from stable COPD individuals compared to healthy smokers	[34]
Q9UK76	HN1	hematological and	Nucleus	other	2.21	NONE	

		neurological expressed 1					
P02647	APOA1	apolipoprotein A-I	Extracellular Space	Lipid metabolism	2.13	COPD biomarker	35
P07108	DBI	diazepam binding inhibitor (GABA receptor modulator, acyl-CoA binding protein)	Cytoplasm	other	2.13	NONE	
P55822	SH3BGR	SH3 domain binding glutamic acid-rich protein	Cytoplasm	other	2.13	NONE	
P08758	ANXA5	annexin A5	Plasma Membrane	Apoptosis pathway	2.1	Decreases macrophage efferocytosis and elastase-induced pulmonary emphysema in mice	36
P37837	TALDO1	transaldolase 1	Cytoplasm	enzyme	2.09	NONE	
P04259	KRT6B	keratin 6B	Cytoplasm	other	2.049	NONE	
P41222	PTGDS	prostaglandin D2 synthase 21kDa (brain)	Cytoplasm	enzyme	2.03	Increased RNA expression in the human lung tissue of	37

						subjects with moderate versus mild COPD	
Q9BWM5	ZNF416	zinc finger protein 416	Nucleus	other	1.98	NONE	
Q9HCE9	ANO8	anoctamin 8	Extracellular Space	other	1.98	NONE	
Q96PP8	GBP5	guanylate binding protein 5	Plasma Membrane	enzyme	1.95	NONE	
Q92888	ARHGEF1	Rho guanine nucleotide exchange factor (GEF) 1	Cytoplasm	other	1.94	NONE	
P51884	LUM	lumican	Extracellular Space	other	1.93	Extracellular matrix component	38
P62937	PPIA	Peptidyl_prolyl cis_trans isomerase A			1.92	Increased in lung tissue from smokers with COPD versus never-smokers, and non-COPD smokers	39
P09972	ALDOC	aldolase C, fructose-bisphosphate	Cytoplasm	Metabolic enzyme	1.91	NONE	
Q5JYT7	KIAA1755	KIAA1755	unknown	other	1.91	NONE	

P30086	PEBP1	phosphatidylethanolamine binding protein 1	Cytoplasm	other	1.9	NONE
O75368	SH3BGRL	SH3 domain binding glutamic acid-rich protein like	Cytoplasm	other	1.89	NONE
Q4G0N8	SLC9C1	solute carrier family 9, subfamily C (Na+-transporting carboxylic acid decarboxylase), member 1	unknown	other	1.89	NONE
O75874	IDH1	isocitrate dehydrogenase 1 (NADP+), soluble	Cytoplasm	enzyme	1.88	NONE
Q13421	MSLN	mesothelin	Extracellular Space	other	1.88	NONE
Q9Y6W5	WASF2	WAS protein family, member 2	Cytoplasm	cytoskeleton	1.87	NONE

P50224	ST1A3	Sulfotransferase 1A3/1A4			1.86	NONE	
Q9Y2K3	MYH15	myosin, heavy chain 15	Extracellular Space	other	1.86	Muscle dysfunction and aberrations of myosin composition within muscle has been associated with COPD	10,11,40-45
Q16881	TXNRD1	thioredoxin reductase 1	Cytoplasm	enzyme	1.82	NONE	
P37802	TAGLN2	transgelin 2	Cytoplasm	other	1.73	NONE	
P35527	KRT9	keratin 9	Cytoplasm	other	1.71	NONE	
P09104	ENO2	enolase 2 (gamma, neuronal)	Cytoplasm	enzyme	1.7	NONE	
P40925	MDH1	malate dehydrogenase 1, NAD (soluble)	Cytoplasm	enzyme	1.68	NONE	

P30041	PRDX6	peroxiredoxin 6	Cytoplasm	enzyme	1.66	NONE	
P04264	K2C1	Keratin type II cytoskeletal 1			1.65	NONE	
P61088	UBE2N	ubiquitin-conjugating enzyme E2N	Cytoplasm	enzyme	1.65	CS induces UBE2N	46
P06319	LV605	Ig lambda chain V_VI region EB4	Extracellular Space	immunoglobin	1.64	NONE	
P20962	PTMS	parathymosin	Nucleus	other	1.63	NONE	
Q8N0Y7	PGAM4	phosphoglycerate mutase family member 4	unknown	phosphatase	1.63	NONE	
P06733	ENO1	enolase 1, (alpha)	Cytoplasm	transcription regulator	1.61	NONE	
P09467	FBP1	fructose-1,6-bisphosphatase 1	Cytoplasm	phosphatase	1.6	NONE	
P17066	HSPA6	heat shock 70kDa protein 6 (HSP70B')	unknown	other	1.59	Increased proteins levels in patients with COPD treated with Inhaled Corticosteroids	47-49
Q96PX6	CCDC85A	coiled-coil domain containing 85A	unknown	other	1.57	NONE	

P23528	CFL1	cofilin 1 (non-muscle)	Nucleus	cytoskeleton	1.56	NONE	
P63261	ACTG	Actin_ cytoplasmic 2	Cytoplasm		1.56	NONE	
P06703	S100A6	S100 calcium binding protein A6	Cytoplasm	transporter	1.55	Calcium binding protein involved in neutrophil activation and protein levels elevated in sputum from COPD versus control subjects	50-52

Table S3: Unique proteins downregulated in BALF (n=138). Differentially expressed proteins with at least 1.5x fold change decrease in the BALF proteome in COPD versus control cohort samples.

Uniprot ID	Symbol	Entrez Gene Name	Location	Type(s)	Fold change	Previously described association with COPD	References
Q9HCH0	NCKAP5L	NCK-associated protein 5-like	unknown	other	-8.55	NONE	
Q01995	TAGLN	transgelin	Cytoplasm	other	-8.11	NONE	
Q29865	HLA-C	major histocompatibility complex, class I, C	Plasma Membrane	other	-7.63	GWAS analysis in the ECLIPSE study noted a SNP in the HLA-C region	53
P14314	PRKCSH	protein kinase C substrate 80K-H	Cytoplasm	enzyme	-6.84	NONE	
B3KS81	SRRM5	serine/arginine repetitive matrix 5	unknown	other	-6.06	NONE	
P20142	PEPC	Gastricsin	Extracellular Space		-5.25	NONE	
O95185	UNC5C	unc-5 homolog C	Plasma Membrane	transmembrane receptor/ netrin	-5.11	NONE	
Q9Y3P9	RABGAP1	RAB GTPase activating protein 1	Cytoplasm	other	-5.04	NONE	

Q7Z3U7	MON2	MON2 homolog (S. cerevisiae)	Cytoplasm	other	-5.02	NONE	
Q9NWN3	FBXO34	F-box protein 34	unknown	other	-4.80	NONE	
O60885	BRD4	bromodomain containing 4	Nucleus	kinase	-4.33	NONE	
Q9UHX3	EMR2	egf-like module containing, Mucin-like, hormone receptor-like 2	Plasma Membrane	G-protein coupled receptor	-4.32	NONE	
Q9NSY1	BMP2K	BMP2 inducible kinase	Nucleus	kinase	-4.03	NONE	
Q9H0P7	CF059	Putative uncharacterized protein encoded by NCRNA00241			-3.96	NONE	
O75419	CDC45	cell division cycle 45 homolog	Nucleus	other	-3.68	NONE	
P03950	ANG	angiogenin, ribonuclease, RNase A family, 5	Extracellular Space	enzyme	-3.59	NONE	
Q8IWL2	SFTPA1	surfactant protein A1	Extracellular Space	transporter	-3.59	Imbalances of the surfactant proteins, major components of alveolar fluid have been implicated in COPD	54-60
Q1ED39	CP088	Protein C16orf88			-3.57	NONE	
P54750	PDE1A	phosphodiesterase 1A, calmodulin-dependent	Cytoplasm	enzyme	-3.48	NONE	

Q8ND24	RNF214	ring finger protein 214	unknown	other	-3.46	NONE	
Q96N16	JAKMIP1	janus kinase and microtubule interacting protein 1	Cytoplasm	other	-3.41	NONE	
Q14980	NUMA1	nuclear mitotic apparatus protein 1	Nucleus	other	-3.26	NONE	
Q9UI36	DACH1	dachshund homolog 1	Nucleus	transcription regulator	-3.03	NONE	
Q9UHG3	PCYOX1	prenylcysteine oxidase 1	Cytoplasm	enzyme	-3.03	NONE	
O14905	WNT9B	wingless-type MMTV integration site family, member 9B	Extracellular Space	Signal transduction	-3.03	NONE	
Q99996	AKAP9	A kinase (PRKA) anchor protein (yotiao) 9	Cytoplasm	other	-3.02	NONE	
Q9Y2P7	ZNF256	zinc finger protein 256	Nucleus	transcription regulator	-3.00	NONE	
P02747	C1QC	complement component 1, q subcomponent, C chain	Extracellular Space	other	-3.00	NONE	
O00750	PIK3C2B	phosphoinositide-3-kinase, class 2, beta polypeptide	Cytoplasm	kinase	-3.00	Associated with glucocorticoid sensitivity and inflammation in COPD	61-63
Q9P2N5	RBM27	RNA binding motif protein 27	Nucleus	other	-3.00	NONE	
Q9Y520	PRRC2C	proline-rich coiled-coil 2C	Cytoplasm	other	-2.92	NONE	

Q9P2Y4	ZNF219	zinc finger protein 219	Nucleus	transcription regulator	-2.92	NONE	
O43813	LANCL1	LanC lantibiotic synthetase component C-like 1 (bacterial)	Plasma Membrane	other	-2.92	NONE	
P01714	LV301	Ig lambda chain V_III region SH	Extracellular Space	immunoglobin	-2.92	NONE	
O75264	CS077	Transmembrane protein C19orf77			-2.92	NONE	
A6NMX2	EIF4E1B	eukaryotic translation initiation factor 4E family member 1B	unknown	other	-2.92	NONE	
Q8IYD8	FANCM	Fanconi anemia, complementation group M	Nucleus	enzyme	-2.92	NONE	
Q8TC84	FANK1	Fibronectin type III and ankyrin repeat domains 1	Nucleus	transcription regulator	-2.92	NONE	
Q96NX9	DACH2	dachshund homolog 2	Nucleus	other	-2.90	NONE	
Q9BVG8	KIFC3	kinesin family member C3	Cytoplasm	enzyme	-2.87	NONE	
O14920	IKBKB	inhibitor of kappa light polypeptide gene enhancer in B-cells, kinase beta	Cytoplasm	kinase	-2.75	Implicated in COPD inflammation	[64-66]
Q92738	USP6NL	USP6 N-terminal like/ RAB5 effector RN-tre	Plasma Membrane	Cytoskeleton element involved in pinocytosis	-2.75	NONE	

Q96JB5	CDK5RAP3	CDK5 regulatory subunit associated protein 3	Cytoplasm	other	-2.74	NONE	
Q9UEW3	MARCO	macrophage receptor with collagenous structure	Plasma Membrane	transmembrane receptor	-2.74	A macrophage scavenger receptor involved in bacterial phagocytosis in COPD	67,68
Q13724	MOGS	mannosyl-oligosaccharide glucosidase	Cytoplasm	enzyme	-2.68	NONE	
P51674	GPM6A	glycoprotein M6A	Plasma Membrane	ion channel	-2.68	NONE	
Q16651	PRSS8	protease, serine, 8	Extracellular Space	peptidase	-2.63	NONE	
O96009	NAPSA	napsin A aspartic peptidase	Extracellular Space	peptidase	-2.58	NONE	
Q9NVX2	NLE1	notchless homolog 1 (Drosophila)	Nucleus	enzyme	-2.54	NONE	
P02751	FN1	fibronectin 1	Extracellular Space	enzyme	-2.53	Matrix protein involved in fibroblast proliferation implicated in COPD pathogenesis	38,69-73

Q13023	AKAP6	A kinase (PRKA) anchor protein 6	Nucleus	other	-2.53	NONE	
Q5VWQ0	RSBN1	round spermatid basic protein 1	Nucleus	other	-2.53	NONE	
Q9UGM5	FETUB	Fetuin B	Extracellular Space	other	-2.49	NONE	
Q9Y2G8	DNAJC16	DnaJ (Hsp40) homolog, subfamily C, member 16	unknown	other	-2.45	NONE	
P35247	SFTPD	surfactant protein D	Extracellular Space	other	-2.44	Imbalances of the surfactant proteins, major components of alveolar fluid have been implicated in COPD	54,56,74-79
Q2TBE0	CWF19L2	CWF19-like 2, cell cycle control	unknown	other	-2.42	NONE	
Q9BYF1	ACE2	angiotensin I converting enzyme (peptidyl-dipeptidase A) 2	Plasma Membrane	peptidase	-2.41		80
O95969	SCGB1D2	secretoglobin, family 1D, member 2	Extracellular Space	other	-2.40	NONE	
P78367	NKX32	Homeobox protein Nkx_3.2			-2.40	NONE	
Q9P275	USP36	ubiquitin specific peptidase 36	Nucleus	peptidase	-2.40	NONE	

O43464	HTRA2	HtrA serine peptidase 2	Cytoplasm	peptidase	-2.40	NONE
O60281	ZNF292	zinc finger protein 292	Nucleus	transcription regulator	-2.38	NONE
Q96JM2	ZNF462	zinc finger protein 462	Nucleus	other	-2.37	NONE
P27487	DPP4	dipeptidyl-peptidase 4	Plasma Membrane	peptidase	-2.33	Putative serum COPD biomarker [81]
Q86SX3	CN080	Uncharacterized protein C14orf80			-2.28	NONE
Q9UJV3	MID2	midline 2	Cytoplasm	other	-2.13	NONE
Q6ZU80	CEP128	centrosomal protein 128kDa	unknown	other	-2.12	NONE
Q01968	OCRL	Inositol polyphosphate 5_phosphatase	Cytoplasm	phosphatase	-2.11	NONE
P42696	RBM34	RNA binding motif protein 34	Nucleus	other	-2.11	NONE

P0CB38	PABPC4L	poly(A) binding protein, cytoplasmic 4-like	unknown	other	-2.09	NONE	
P49770	EIF2B2	eukaryotic translation initiation factor 2B, subunit 2 beta, 39kDa	Cytoplasm	translation regulator	-2.09	NONE	
P11678	EPX	eosinophil peroxidase	Cytoplasm	enzyme	-2.06	NONE, Although one reference examined EPX in subjects, it is was NOT differentially expressed in COPD	82
B1AJZ9	FHAD1	forkhead-associated (FHA) phosphopeptide binding domain 1	unknown	other	-2.04	NONE	

P54920	NAPA	N-ethylmaleimide-sensitive factor attachment protein, alpha	Cytoplasm	other	-2.03	NONE
P01771	HV310	Ig heavy chain V_III region HIL	Extracellular Space	immunoglobin	-2.03	NONE
Q9Y2H0	DLGAP4	discs, large homolog-associated protein 4	Plasma Membrane	other	-2.01	NONE
Q07954	LRP1 (includes EG:16971)	low density lipoprotein receptor-related protein 1	Plasma Membrane	transmembrane receptor	-2.00	NONE

Q13702	RAPSN	receptor-associated protein of the synapse	Plasma Membrane	other	-2.00	NONE
Q13620	CUL4B	cullin 4B	Nucleus	other	-2.00	NONE
P15291	B4GALT1	UDP-Gal:betaGlcNAc beta 1,4- galactosyltransferase, polypeptide 1	Cytoplasm	enzyme	-1.99	NONE
P04259	K2C6B	Keratin type II cytoskeletal 6B			-1.99	NONE
P14384	CPM	carboxypeptidase M	Plasma Membrane	peptidase	-1.98	NONE
Q5HYK9	ZNF667	zinc finger protein 667	Nucleus	other	-1.97	NONE
Q5VVM6	CCDC30	coiled-coil domain containing 30	unknown	other	-1.97	NONE

Q16181	SEP7	septin 7	Cytoplasm	other	-1.97	NONE
P01763	HV302	Ig heavy chain V_III region WEA	Extracellular Space	immunoglobin	-1.96	NONE
P07225	PROS1	protein S (alpha)	Extracellular Space	other	-1.96	NONE
P01780	HV319	Ig heavy chain V_III region JON	Extracellular Space	immunoglobin	-1.95	NONE
P01605	KV113	Ig kappa chain V_I region Lay	Extracellular Space	immunoglobin	-1.95	NONE

P01611	KV119	Ig kappa chain V_I region Wes	Extracellular Space	immunoglobin	-1.93	NONE
P01612	KV120	Ig kappa chain V_I region Mev	Extracellular Space	immunoglobin	-1.93	NONE
Q8NB66	UNC13C	unc-13 homolog C	Cytoplasm	other	-1.90	NONE
A4D1S5	RAB19	RAB19, member RAS oncogene family	Cytoplasm	enzyme	-1.90	NONE
Q9Y613	FHOD1	formin homology 2 domain containing 1	Nucleus	other	-1.89	NONE
Q86Y33	CDC20B	cell division cycle 20 homolog B	unknown	other	-1.87	NONE

P06317	LV603	Ig lambda chain V_VI region SUT	Extracellular Space	immunoglobin	-1.86	NONE
Q5FWF4	ZRANB3	zinc finger, RAN-binding domain containing 3	unknown	enzyme	-1.83	NONE
Q0VAM2	RASGEF1B	RasGEF domain family, member 1B	unknown	other	-1.82	NONE
Q9HAV0	GNB4	guanine nucleotide binding protein (G protein), beta polypeptide 4	Plasma Membrane	enzyme	-1.79	NONE
P16070	CD44	CD44 molecule (Indian blood group)	Plasma Membrane	enzyme	-1.79	CD44 HA receptor is implicated in macrophage phagocytic ability [9,83-86]

						and appears to be decreased in COPD	
P16144	ITGB4	integrin, beta 4	Plasma Membrane	transmembrane receptor	-1.77	NONE	
Q12873	CHD3	chromodomain helicase DNA binding protein 3	Nucleus	enzyme	-1.75	NONE	
P15151	PVR	poliovirus receptor	Plasma Membrane	other	-1.75	NONE	
Q08380	LGALS3BP	lectin, galactoside-binding, soluble, 3 binding protein	Plasma Membrane	transmembrane receptor	-1.75	NONE for the specific protein, but as a binding protein for a lectin, its associates with	[26,27]

						galectin 3 which seems to regulate macrophage efferocytosis in COPD, ,
Q9HBR0	SLC38A10	solute carrier family 38, member 10	unknown	other	-1.74	NONE
Q6Q759	SPAG17	sperm associated antigen 17	unknown	other	-1.73	NONE
Q15582	TGFBI	transforming growth factor, beta-induced, 68kDa	Extracellular Space	other	-1.73	NONE
A6NCL7	ANKRD33B	ankyrin repeat domain 33B	unknown	other	-1.72	NONE

Q8TEU7	RAPGEF6	Rap guanine nucleotide exchange factor (GEF) 6	Plasma Membrane	other	-1.72	NONE
P01619	KV301	Ig kappa chain V_III region B6	Extracellular Space	immunoglobin	-1.72	NONE
Q8TCU6	PREX1	phosphatidylinositol-3,4,5-trisphosphate-dependent Rac exchange factor 1	Cytoplasm	other	-1.70	NONE
P04114	APOB	apolipoprotein B (including Ag(x) antigen)	Extracellular Space	Lipid metabolism	-1.68	NONE
P06314	KV40	Ig kappa chain V_IV region B17	Extracellular Space	immunoglobin	-1.67	NONE

P01859	IGHG2	immunoglobulin heavy constant gamma 2 (G2m marker)	Plasma Membrane	immunoglobin	-1.67	NONE
P17174	GOT1	glutamic-oxaloacetic transaminase 1, soluble (aspartate aminotransferase 1)	Cytoplasm	enzyme	-1.67	NONE
Q02818	NUCB1	nucleobindin 1	Cytoplasm	other	-1.67	NONE
P05362	ICAM1	intercellular adhesion molecule 1	Plasma Membrane	transmembrane receptor	-1.66	Discordant of ICAM in COPD in literature compared to our findings [87-96]

Q6UX72	B3GNT9	UDP-GlcNAc:betaGal beta-1,3-N-acetylglucosaminyltransferase 9	unknown	enzyme	-1.65	NONE	
P52823	STC1	stanniocalcin 1	Extracellular Space	kinase	-1.65	NONE	
P36222	CHI3L1	chitinase 3-like 1	Extracellular Space	Tissue remodeling	-1.65	Increased in the serum and BAL of smokers with COPD compared to never smokers or smokers without COPD	97-99

Q96JH7	VCPIP1	valosin containing protein (p97)/p47 complex interacting protein 1	Cytoplasm	peptidase	-1.64	NONE	
O95436	SLC34A2	solute carrier family 34 (sodium phosphate), member 2	Plasma Membrane	transporter	-1.64	NONE	
P46199	MTIF2	mitochondrial translational initiation factor 2	Cytoplasm	translation regulator	-1.64	NONE	
Q86SQ7	SDCCAG8	serologically defined colon cancer antigen 8	Cytoplasm	other	-1.64	NONE	
Q8N7W2	BEND7	BEN domain containing 7	unknown	other	-1.61	NONE	
P01024	C3	complement component 3	Extracellular Space	peptidase	-1.58	Serum levels of C3 was decreased in serum and sputum	[100-102]

						of subjects with COPD	
P07998	RNASE1	ribonuclease, RNase A family, 1 (pancreatic)	Extracellular Space	enzyme	-1.58	NONE	
P00739	HPR	haptoglobin-related protein	Extracellular Space	peptidase	-1.58	NONE	
Q76L83	ASXL2	additional sex combs like 2	Extracellular Space	other	-1.57	NONE	
Q8NFJ5	GPRC5A	G protein-coupled receptor, family C, group 5, member A	Plasma Membrane	G-protein coupled receptor	-1.57	Decreased in lung epithelia associated with lung adenocarcinoma	[103,104]

						compared to epithelia from subjects with COPD or never smokers	
Q7Z7M9	GALNT5	UDP-N-acetyl-alpha-D-galactosamine:polypeptide N-acetylgalactosaminyltransferase 5 (GalNAc-T5)	Cytoplasm	enzyme	-1.57	NONE	
P06331	HV209	Ig heavy chain V_II region ARH_77	Extracellular Space	immunoglobin	-1.57	NONE	
P15941	MUC1	Mucin 1, cell surface associated	Plasma Membrane	transcription regulator	-1.56	Levels are affected by age and smoking in lung tissue, sputum and plasma	56,105,106

P48960	CD97	CD97 molecule	Plasma Membrane	G-protein coupled receptor	-1.55	NONE	
P06681	C2	complement component 2	Extracellular Space	peptidase	-1.54	NONE	
Q9NYQ6	CELSR1	cadherin, EGF LAG seven-pass G-type receptor 1	Plasma Membrane	G-protein coupled receptor	-1.53	NONE	
P0CG06	IGLC3	immunoglobulin lambda constant 3 (Kern-Oz+ marker)	Extracellular Space	other	-1.53	NONE	
P06126	CD1A	CD1a molecule	Plasma Membrane	other	-1.52		107-110

O00560	SDCBP	syndecan binding protein (syntenin)	Plasma Membrane	enzyme	-1.51	NONE

Table S4:

See David_FuncAnnotClustering_BALF.csv for DAVID functional annotation clustering file.

Table S5:

Transcription factors associated with binding sites on genes from differentially expressed proteins in BALF. Transcription factors that have association with binding sites on genes from differentially expressed proteins in BALF as noted by DAVID [ref].

Transcription factor with binding sites in the genes represented in BALF	Number of the differentially expressed proteins that have corresponding gene binding sites with transcription factor	% of dataset in DAVID database	p-value (Fisher exact)
AREB6	166	79.43	0.009
SRF	148	70.81	0.000
AML1	147	70.33	0.089
P53	127	60.77	0.061
AP4	125	59.81	0.053

LMO2COM	122	58.37	0.004
SREBP1	118	56.46	0.046
PAX2	113	54.07	0.073
USF	113	54.07	0.081
PAX5	112	53.59	0.034
GCNF	112	53.59	0.049
STAT5A	111	53.11	0.074
MRF2	110	52.63	0.001
HTF	110	52.63	0.010
TAXCREB	109	52.15	0.021
CEBPB	109	52.15	0.041
AHRARNT	105	50.24	0.031
FOXO4	105	50.24	0.073
RP58	104	49.76	0.032
STAT3	101	48.33	0.004
BACH1	101	48.33	0.028
HNF4	100	47.85	0.010
GFI1	97	46.41	0.002
NFKB	97	46.41	0.071
STAT1	96	45.93	0.000
FREAC3	95	45.45	0.001
CREBP1	95	45.45	0.033
OCT	94	44.98	0.025
CDPCR3HD	92	44.02	0.019
HAND1E47	92	44.02	0.052
RSRFC4	91	43.54	0.091
SOX9	90	43.06	0.028
HFH1	90	43.06	0.044
CMYB	89	42.58	0.014
GATA	89	42.58	0.050
HSF2	88	42.11	0.012
IK3	88	42.11	0.058
HOX13	88	42.11	0.098

RORA1	87	41.63	0.087
HLF	86	41.15	0.009
FOXO1	85	40.67	0.026
TGIF	84	40.19	0.060
POU6F1	84	40.19	0.068
E4BP4	84	40.19	0.100
MIF1	83	39.71	0.074
STAT	82	39.23	0.001
CP2	82	39.23	0.020
MSX1	81	38.76	0.098
LYF1	80	38.28	0.032
HNF3B	80	38.28	0.091
NFKAPPAB	79	37.80	0.004
NFE2	70	33.49	0.094
FOXD3	69	33.01	0.061
IK2	62	29.67	0.054
TAL1BETAE47	61	29.19	0.041
HSF1	61	29.19	0.087
ZIC2	53	25.36	0.016
GATA3	51	24.40	0.034
MAX	37	17.70	0.089

Table S6: Top functional networks of differentially expressed molecules in the BALF proteome. The top biological functions associated with molecular pathways imputed with IPA that are significantly associated differentially expressed molecules measured in the BALF proteome. Red represents upregulated, and green represents downregulated proteins. The networks are collections of interconnected molecules assembled by a network algorithm. Each connection represents known relationships between the molecules, found in the Ingenuity Knowledge Base. The score is the degree of relevance of network eligible molecules to the BALF dataset. The

score takes into account the number of network eligible molecules in the network and its size, as well as the total number of network eligible molecules analyzed and the total number of molecules in the Ingenuity Knowledge Base that could potentially be included in networks. The network score is based on the hypergeometric distribution and is calculated with the right-tailed Fisher's Exact Test: Score=-log(Fisher's Exact test result). Focus Molecules are the number of proteins identified in the BALF proteome that is found in the network.

**ID**	Molecules in network	Score	Focus molecules	Top biological functions associated with the molecular network
**1**	ACTG1, Actin, Akt, Alpha catenin, ANXA5, ARHGEF1, CD44, CFL1, Collagen type I, COTL1, F Actin, FLNA, HN1, Hsp27, ITGB4, Laminin, LGALS3, LUM, MARK2, MUC1, MYH11, MYO7A, PLEC, PRKCSH, PROS1, PTGDS, PVR, Rock, SDCBP, TGFBI, TMSB10/TMSB4X, TXNRD1, USP6NL, VIM, WASF2	51	27	Cellular Movement, Inflammatory Response, Cardiovascular System Development and Function
**2**	AKR1C3, ANG, APCS, C3, C1q, C1QC, C4BP, CD1A, CHI3L1, Complement component 1, ENO1, EPX, ERK1/2, ETS, Fcer1, FETUB, FHOD1, Gm-csf, Ige, LAMA3, LRP1, Mac1, MARCO, PIK3C2B, PLA2, PLA2G1B, PPIA, PPIB, PRDX6, Rsk, S100A6, Sos, STC1, T3-TR-RXR, TH2 Cytokine	36	21	Cell Death and Survival, Drug Metabolism, Small Molecule Biochemistry
**3**	APC, BOD1L1, C10orf116, C14orf80, C5orf51, CDC37, CEP128, CUL2, DDIT3, ELAVL1, GSK3B, GSTP1, KIAA0101, MYH15, NCKAP5L, PABPC4L, PCNA, RBM27, RNF214, RPS6KA6, RSBN1, SCGB1D2, SLC38A10, SND1, TRIM28, UBC, VAV2, ZNF256, ZNF667, ZRANB3	27	16	Cell Morphology, Cellular Assembly and Organization, Cellular Development
**4**	A2M, APOA1, APOB, APOC3, B3GNT9, chymotrypsin, Cytokeratin, elastase, FGA, FGB, FGG, Fibrin, Fibrinogen, GPIIB-IIIA, Growth hormone, HDL, HDL-cholesterol, HP, HPR, Kallikrein, KRT1, KRT9, KRT10, KRT6B, LDL-cholesterol, LRP, NFkB (complex), PCYOX1, PEBP1, Pro-inflammatory Cytokine, SAA, SFTPA1, SFTPD, Stat3-Stat3, VLDL-cholesterol	27	18	Developmental Disorder, Hematological Disease, Hereditary Disorder

**5**	ANXA3, APITD1, C1GALT1C1, C1orf86, C9orf72, CYC1, EIF2B2, EIF2B3, EMG1, FANCB, FANCE, FANCF, FANCM, GALNT2, GALNT5, GGA1, GGA3, KRT79, LANCL1, MON2, MYOZ1, NAPSA, NLE1, PNKP, RAB6B, RABGAP1, RAPGEF6, RBM34, RMI2, SH3BGRL, SLC25A24, STRA13, TOP3A, UBC, ZNF292	25	16	Developmental Disorder, Hematological Disease, Hereditary Disorder
**6**	Alp, BMP2K, C16orf88, CBR1, CD3, CDC45, Cg, CHD3, CNGA2, CUL4B, DBI, ENO2, Focal adhesion kinase, Hdac, Histone h3, Histone h4, Hsp70, HSPA6, ICAM1, IDH1, IKK (complex), LDL, NADPH oxidase, P38 MAPK, Pdgf (complex), PI3K (complex), Pkc(s), PREX1, RNA polymerase II, Sod, SRC (family), TALDO1, Vegf, VNN1, WNT9B	24	16	Cancer, Gastrointestinal Disease, Cardiovascular Disease
**7**	AKAP6, BEND7, C11orf48, CCDC85A, CCNB1, CCND1, CDK5RAP3, CDKN1B, CMIP, DACH2, DDRGK1, DGCR14, DNAJC16, FOXO3, GSTM4, GSTM5, GSTO2, hemoglobin, LYAR, MYRIP, NANOG, PARPBP, PGAM4, PIK3R1, PRRC2C, RAB19, RSL24D1, SIX6, SLC34A2, STAT5A, TMEM55A, UBC, UBLCP1, UFC1, ZNF462	20	14	Cardiovascular System Development and Function, Cell Cycle, Skeletal and Muscular System Development and Function
**8**	ADCYAP1, alcohol dehydrogenase, ALDH16A1, ALDOC, APP, ASXL2, C19orf40, CALML3, CASP6, CCL5, CRTAC1, CWF19L2, FBXO34, GSTM3, HSP90AB1, HSPA2, HSPB7, IRAK3, KIFC3, MDH1, NUCB1, PDE1A, PSMB4, PSMD1, PTMS, RAB10, RNASE1, RUSC1, SCAVENGER receptor CLASS A, SDCCAG8, SH3RF2, TAGLN2, TRAF6, USP1, ZBTB20	20	14	Organismal Injury and Abnormalities, Cell Death and Survival, Nervous System Development and Function
**9**	B4GALT1, BCR (complex), Collagen(s), CTSZ, DPP4, ERK, Fc gamma receptor, GATA3, GOT1, HLA-C, HSP, Ifn, IFN Beta, Ifn gamma, IgG1, Igg3, IgG, IGHG2, Igm, Ikb, IKBKB, IL1, IL12 (complex), IL12 (family), Immunoglobulin, Interferon alpha, LGALS3BP, MHC Class I (complex), MHC CLASS I (family), MHC Class II (complex), NKX3-2, PPBP, PRSS8, Tgf beta, Tlr	17	12	Cellular Movement, Hematological System Development and Function, Immune Cell Trafficking
**10**	26sProteasome, ADCY, ARHGAP24, Calmodulin, CD97, CELSR1, chemokine, Ck2, EMR2, endocannabinoid, FBP1, FSH, Gpcr, GPR4, GPR68, GPRC5A, GRM8, Insulin, MAP9, Mapk, MID2, NAPA, OCRL, Pka, PLC, Rac, RAPSN, Ras, Ras homolog, Sfk, Shc, SYTL4, Trk Receptor, UBE2N, Ubiquitin	17	12	Cellular Assembly and Organization, Cellular Function and Maintenance, Molecular Transport

**11**	ADH1B, AKAP9, ALDH3A1, Ap1, BLVRA, C2, C/ebp, calpain, caspase, Collagen type IV, Cyclin A, Cyclin E, DACH1, DLGAP4, estrogen receptor, FHL1, FN1, Hsp90, HTRA2, Integrin, Jnk, Lfa-1, MAP2K1/2, Mek, Metalloprotease, Mmp, MSLN, NFAT (complex), Nfat (family), NUMA1, p70 S6k, PDGF BB, PSMD14, TCR, trypsin	16	13	Cell Cycle, Visual System Development and Function, Hair and Skin Development and Function
**12**	ADRBK2, CACNA1B, CCM2, CHRM3, CNR1, COL11A2, COL2A1, CREB3L3, D-glucose, endocannabinoid, FCHSD2, GABBR1, GBP5, GNB4, GNG5, GNG7, GNGT1, GPM6A, GPR68, ITGB1BP1, JAKMIP1, KRIT1, N-type Calcium Channel, PLA2G6, PLCB3, RGS6, SEPT4, SEPT7, SEPT8, SH3BGR, TRHR, TRPV4, UNC13C, VCPIP1, ZNF219	13	10	Connective Tissue Disorders, Developmental Disorder, Hereditary Disorder
**13**	ACP5, AKAP12, BCL3, CAMP, CPM, CSF1, CYP11A1, FANK1, FPR2, Hedgehog, HLX, HSD17B1, ITGB8, JUN, mannitol, MAP2K2, MAZ, MOGS, MTIF2, NOTCH4, PGC, PROM1, PTPRO, SERPINB2, SFTPB, SIRT6, SLC8A1, SMAD5, SOD2, STAB2, Stat3-Stat3, TEAD4, TMSB10/TMSB4X, USP36, VEGFA	8	7	Cardiovascular System Development and Function, Embryonic Development, Organismal Development
**14**	ANO8, COQ9	2	1	Hereditary Disorder, Metabolic Disease, Cancer
**15**	Spag6, SPAG17	2	1	Cellular Assembly and Organization, Cellular Compromise, Cellular Function and Maintenance
**16**	ADCY10, SLC9C1	2	1	Cellular Movement, Reproductive System Development and Function, Reproductive System Disease

Red=upregulated proteins

Green=downregulated protein

# Networks = collections of interconnected molecules assembled by a network algorithm. Each connection represents known relationships between the molecules, found in the Ingenuity Knowledge Base.

* Score= The degree of relevance of Network Eligible molecules to the BALF dataset. The score takes into account the number of Network Eligible molecules in the network and its size, as well as the total number of Network Eligible molecules analyzed and the total number of molecules in the Ingenuity Knowledge Base that could potentially be included in networks. The network Score is based on the hypergeometric distribution and is calculated with the right-tailed Fisher's Exact Test. Score=-log(Fisher's Exact test result)

^ Focus Molecules= The number of proteins identified in the BALF proteome that is found in the network

TABLE S7

Computational drug prediction CANDO

(score1= refers to the consensus score or number of times the compound shows up in the top 30 most similar drugs used to treat COPD
score2= the average of the ranks for 'score1'
probability= the binomial distribution derived probability of achieving 'score1' by chance based on the number of drugs associated with COPD, the total number of drugs in the library, and the number of most similar drugs to consider (in this case, 30).
name= generic name of the candidate drug)
CANDO= Computational Analysis of Novel Drug Opportunities
COPD= chronic obstructive pulmonary disease

Rank	Score1 (consensus score)	Score2 (average ranks score)	Probability	Name
1	12	10.1	1.11E-16	clobetasol_propionate
2	12	11.8	1.11E-16	clobetasol

3	12	12.7	1.11E-16	rimexolone
4	11	12.5	4.88E-15	deflazacort
5	10	7.2	2.07E-13	loteprednol_etabonate
6	10	10.4	2.07E-13	desoximetasone
7	10	13.1	2.07E-13	loteprednol
8	10	13.4	2.07E-13	meprednisone
9	10	14.4	2.07E-13	amcinonide
10	9	13.2	7.66E-12	fluclorolone_acetonide
11	9	15.1	7.66E-12	fluorometholone
12	9	17.7	7.66E-12	clobetasone
13	8	14.2	2.48E-10	ulobetasol
14	8	16.1	2.48E-10	procaterol
15	8	18.1	2.48E-10	desonide
16	7	11	6.95E-09	prednicarbate
17	7	11.6	6.95E-09	tezacaftor
18	7	16	6.95E-09	cyproterone_acetate
19	7	24.3	6.95E-09	drometrizole_trisiloxane

20	6	14.2	1.67E-07	nadolol
21	6	14.5	1.67E-07	hydrocortamate
22	6	19.7	1.67E-07	hydrocortisone_butyrate
23	6	23.2	1.67E-07	flurandrenolide
24	5	3	3.39E-06	isoprenaline
25	5	4.6	3.39E-06	epinephrine
26	5	5.6	3.39E-06	orciprenaline
27	5	6.6	3.39E-06	isoetharine
28	5	7.6	3.39E-06	carbidopa
29	5	9.2	3.39E-06	gemfibrozil
30	5	9.8	3.39E-06	phenylephrine
31	5	9.8	3.39E-06	methyldopa
32	5	10.8	3.39E-06	carteolol
33	5	11.2	3.39E-06	paramethasone_acetate
34	5	11.8	3.39E-06	clocortolone
35	5	12.2	3.39E-06	arbutamine
36	5	12.4	3.39E-06	pindolol

37	5	13.4	3.39E-06	hydrocortisone_acetate
38	5	13.8	3.39E-06	levobunolol
39	5	14.6	3.39E-06	difluocortolone
40	5	17.6	3.39E-06	propofol
41	5	18.6	3.39E-06	celiprolol
42	5	18.8	3.39E-06	levonordefrin
43	5	20.8	3.39E-06	tapentadol
44	5	20.8	3.39E-06	segesterone_acetate
45	4	9.2	5.72E-05	betamethasone
46	4	9.2	5.72E-05	methylprednisolone_aceponate
47	4	10.2	5.72E-05	dexamethasone
48	4	11	5.72E-05	naldemedine
49	4	11.2	5.72E-05	elvitegravir
50	4	14	5.72E-05	difluprednate
51	4	15.8	5.72E-05	deferiprone

52	4	17	5.72E-05	methylprednisolone
53	4	17.5	5.72E-05	prednisolone
54	4	18	5.72E-05	tamsulosin
55	4	20.5	5.72E-05	fluprednisolone
56	4	22.2	5.72E-05	canrenoic_acid
57	4	22.5	5.72E-05	etidocaine
58	4	23	5.72E-05	mephenesin
59	4	24.5	5.72E-05	hydrocortisone_valerate
60	4	25.5	5.72E-05	halcinonide
61	4	26	5.72E-05	desvenlafaxine
62	3	5	7.80E-04	fexofenadine
63	3	7	7.80E-04	pioglitazone
64	3	7.7	7.80E-04	laropiprant
65	3	8.3	7.80E-04	terfenadine
66	3	8.7	7.80E-04	cyclandelate

67	3	9.7	7.80E-04	dobutamine
68	3	10	7.80E-04	mometasone_furoate
69	3	10.3	7.80E-04	fentanyl
70	3	11	7.80E-04	amiodarone
71	3	11.3	7.80E-04	siponimod
72	3	12.3	7.80E-04	homatropine_methylbromide
73	3	14	7.80E-04	metaraminol
74	3	14	7.80E-04	flunisolide
75	3	14.3	7.80E-04	meradimate
76	3	14.7	7.80E-04	fluocinonide
77	3	15	7.80E-04	masoprocol
78	3	15	7.80E-04	fluocinolone_acetonide
79	3	15.3	7.80E-04	loperamide
80	3	16	7.80E-04	hydrocortisone_cypionate
81	3	16	7.80E-04	piritramide

82	3	16.3	7.80E-04	darifenacin
83	3	17	7.80E-04	ebastine
84	3	18	7.80E-04	guaifenesin
85	3	18.7	7.80E-04	zolmitriptan
86	3	19	7.80E-04	trospium
87	3	19.3	7.80E-04	darolutamide
88	3	19.7	7.80E-04	olmesartan
89	3	20.3	7.80E-04	megestrol_acetate
90	3	20.7	7.80E-04	levocabastine
91	3	23	7.80E-04	benserazide
92	3	26	7.80E-04	stiripentol
93	3	27	7.80E-04	dipivefrin
94	3	29	7.80E-04	norepinephrine
95	2	1	8.29E-03	oxtriphylline
96	2	2	8.29E-03	bromotheophylline
97	2	2	8.29E-03	methscopolamine_bromide

98	2	2	8.29E-03	butylscopolamine
99	2	2.5	8.29E-03	diethylamino_hydroxybenzoyl_hexyl_benzoate
100	2	3	8.29E-03	caffeine
101	2	3	8.29E-03	methscopolamine
102	2	4	8.29E-03	xanthinol
103	2	4	8.29E-03	dopexamine
104	2	4.5	8.29E-03	difenoxin
105	2	4.5	8.29E-03	scopolamine
106	2	5	8.29E-03	enprofylline
107	2	5	8.29E-03	oxyphenonium
108	2	5.5	8.29E-03	cortisone_acetate
109	2	5.5	8.29E-03	labetalol
110	2	6	8.29E-03	pentoxifylline
111	2	6	8.29E-03	oxybutynin
112	2	7	8.29E-03	dyphylline

113	2	7	8.29E-03	methylphenidate
114	2	8	8.29E-03	temozolomide
115	2	8	8.29E-03	cyclopentolate
116	2	8	8.29E-03	dexmethylphenidate
117	2	9	8.29E-03	enoxacin
118	2	9	8.29E-03	lemborexant
119	2	9	8.29E-03	diflorasone
120	2	9	8.29E-03	fluocortolone
121	2	9.5	8.29E-03	apremilast
122	2	9.5	8.29E-03	mepenzolate
123	2	9.5	8.29E-03	mebeverine
124	2	9.5	8.29E-03	etofamide
125	2	10	8.29E-03	tipiracil
126	2	10	8.29E-03	flumethasone
127	2	10.5	8.29E-03	diphenoxylate
128	2	10.5	8.29E-03	ambenonium

129	2	12	8.29E-03	dexrazoxane
130	2	12	8.29E-03	rosiglitazone
131	2	12.5	8.29E-03	halofantrine
132	2	13	8.29E-03	daunorubicin
133	2	13	8.29E-03	dicloxacillin
134	2	13.5	8.29E-03	permethrin
135	2	13.5	8.29E-03	trimethaphan
136	2	14	8.29E-03	tinidazole
137	2	14	8.29E-03	penbutolol
138	2	14	8.29E-03	oxyphencyclimine
139	2	15	8.29E-03	acetazolamide
140	2	15	8.29E-03	methylergometrine
141	2	15	8.29E-03	cloxacillin
142	2	15	8.29E-03	ecamsule
143	2	15	8.29E-03	sonidegib
144	2	15.5	8.29E-03	cefpirome
145	2	15.5	8.29E-03	zofenopril

146	2	15.5	8.29E-03	panobinostat
147	2	16	8.29E-03	methimazole
148	2	16.5	8.29E-03	troglitazone
149	2	16.5	8.29E-03	flucloxacillin
150	2	17	8.29E-03	levofloxacin
151	2	17	8.29E-03	sumatriptan
152	2	17	8.29E-03	pentoxyverine
153	2	17.5	8.29E-03	elagolix
154	2	17.5	8.29E-03	hexylcaine
155	2	18	8.29E-03	ofloxacin
156	2	18	8.29E-03	alclometasone
157	2	19	8.29E-03	lomefloxacin
158	2	19	8.29E-03	ioflupane_i-123
159	2	19	8.29E-03	losartan
160	2	19.5	8.29E-03	bemotrizinol
161	2	20	8.29E-03	epirubicin

162	2	20	8.29E-03	benzethonium
163	2	20	8.29E-03	benazepril
164	2	20	8.29E-03	mepyramine
165	2	20.5	8.29E-03	maraviroc
166	2	20.5	8.29E-03	methysergide
167	2	20.5	8.29E-03	dextropropoxyphene
168	2	20.5	8.29E-03	ritodrine
169	2	21	8.29E-03	doxorubicin
170	2	21	8.29E-03	cinchocaine
171	2	21	8.29E-03	cefapirin
172	2	21.5	8.29E-03	tegaserod
173	2	21.5	8.29E-03	nomegestrol
174	2	22	8.29E-03	dacarbazine
175	2	22.5	8.29E-03	oxeladin
176	2	23	8.29E-03	deutetrabenazine
177	2	23	8.29E-03	tropicamide
178	2	23	8.29E-03	triamcinolone

179	2	23.5	8.29E-03	lovastatin
180	2	24	8.29E-03	methazolamide
181	2	25	8.29E-03	pefloxacin
182	2	26	8.29E-03	nalidixic_acid
183	2	26	8.29E-03	norgestimate
184	2	26.5	8.29E-03	gestrinone
185	2	26.5	8.29E-03	ergometrine
186	2	27	8.29E-03	dorzolamide
187	2	27	8.29E-03	ethylhexyl_methoxycrylene
188	2	28	8.29E-03	lenalidomide
189	2	29	8.29E-03	idarubicin

Table S8

Predicted interactions of drugs treating respiratory diseases and central node proteins

Representative drugs treating respiratory disease from selected categories showing their predicted interactions with the most central nodes entities of Figure 4

**Drug**	**Category**	**Undesired effect**	**Desired effect**
azithromycin	antibiotic with anti-inflammatory effects	VIM, ICAM1	FN1
BIBF 1120	anti-fibrotic agent	FN1	--
fluticasone	inhaled corticosteroid	ICAM1, CD44	--
pirfenidone	anti-fibrotic agent	FN1, ICAM1	VIM
roflumilast	phosphodiesterase inhibitor	ICAM1, FN1	VIM
salbutamol	short acting beta agonist	FN1	--
salmeterol	long-acting beta agonist	CD44	--
tiotropium	long-acting anti-muscarinic agent	FN1, ICAM1	--

Supplemental Figures

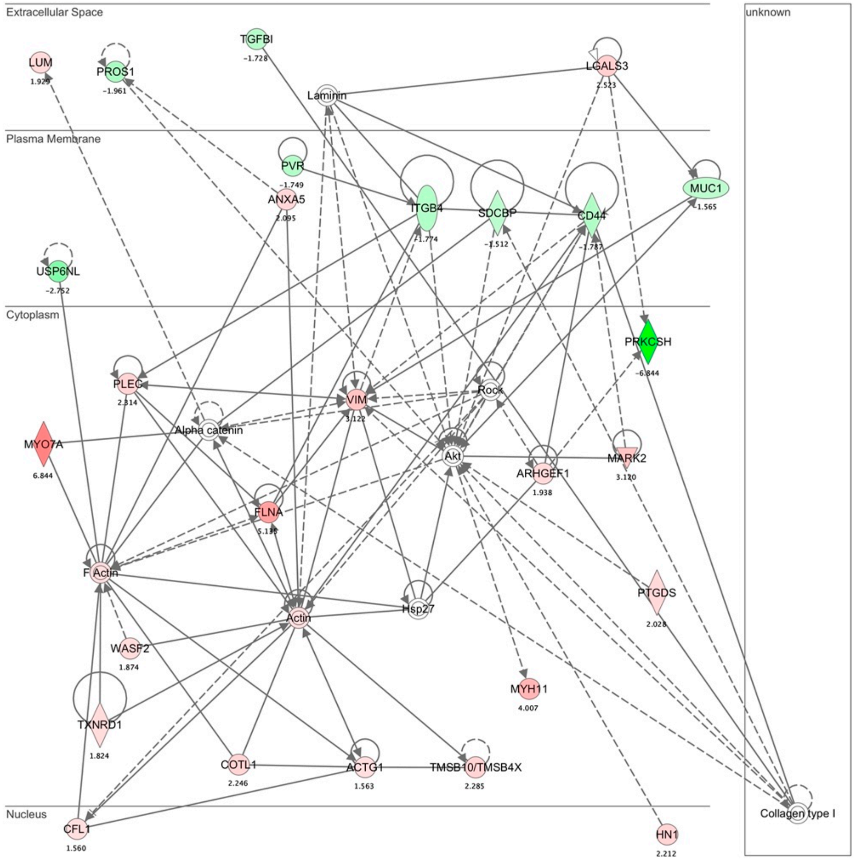

Figure S1:

IPA Network 1: Cellular Movement, Inflammatory Response, Cardiovascular System Development and Function

Functional annotation networks from IPA show relationships among the genes that in IPA's relational database are related to cellular movement, inflammatory response and cardiovascular system development and function.

Solid lines indicated a direct interaction between proteins, while dotted lines indicate an indirect association between two proteins. Proteins upregulated in the BALF dataset are shaded in red and proteins downregulated in the BALF dataset are shaded in green. The darker shading to lighter shading corresponds to decreasing expression intensity.

IPA=Ingenuity Pathway Analysis, BALF= Bronchoalveolar Lavage Fluid

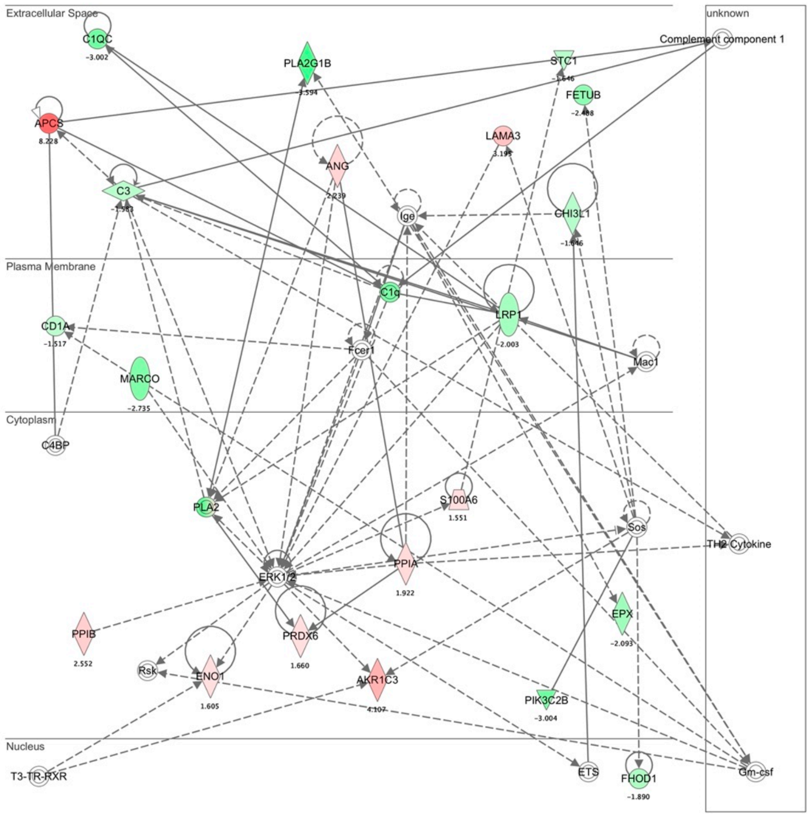

Figure S2:

IPA network 2: cell death and survival, drug metabolism, small molecule biochemistry

Functional annotation networks from IPA that show relationships among the genes that in IPA's relational database are related to cell death and survival, drug metabolism and small molecule biochemistry.

Solid lines indicated a direct interaction between proteins, while dotted lines indicate an indirect association between two proteins. Proteins upregulated in the BALF dataset are shaded in red and proteins downregulated in the BALF dataset are shaded in green. The darker shading to lighter shading corresponds to decreasing expression intensity.

IPA=Ingenuity Pathway Analysis, BALF= Bronchoalveolar Lavage Fluid

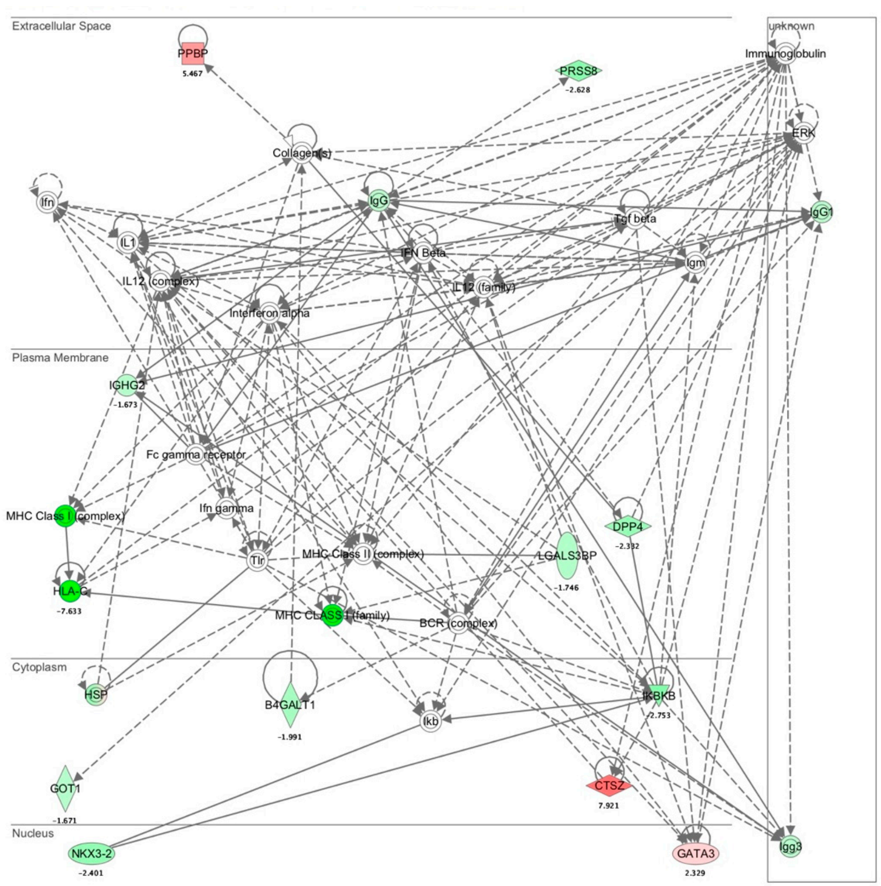

Figure S3:

IPA network 9: cellular movement, hematological system development and function, immune cell trafficking

Functional annotation networks from IPA that show relationships among the genes that in IPA’s relational database are related to cellular movement, hematological system development and function, and immune cell trafficking.

Solid lines indicated a direct interaction between proteins, while dotted lines indicate an indirect association between two proteins. Proteins upregulated in the BALF dataset are shaded in red and proteins downregulated in the BALF dataset are shaded in green. The darker shading to lighter shading corresponds to decreasing expression intensity.

IPA=Ingenuity Pathway Analysis, BALF= Bronchoalveolar Lavage Fluid

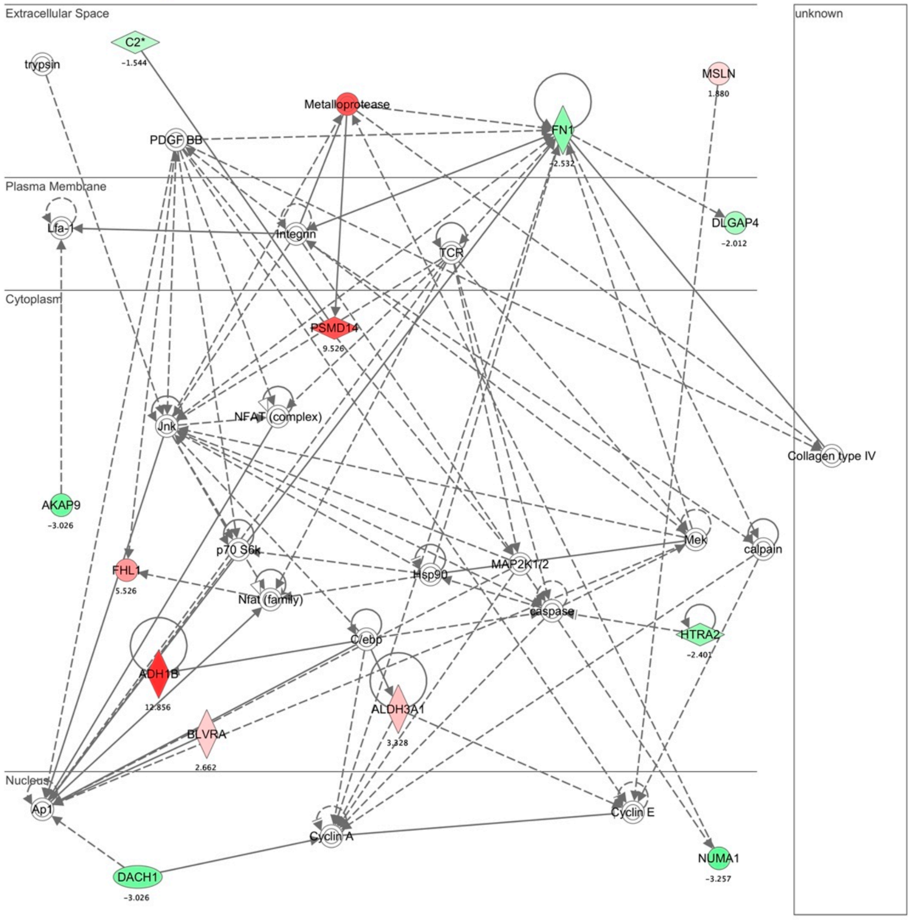

Extracellular Space
unknown
C2*
−1.544
trypsin
MSLN
1.880
Metalloprotease
FN1
−2.532
PDGF BB
Plasma Membrane
Lfa-1
Integrin
DLGAP4
−2.012
TCR
Cytoplasm
PSMD14
9.526
Jnk
NFAT (complex)
Collagen type IV
AKAP9
−3.026
Mek
calpain
p70 S6k
MAP2K1/2
FHL1
5.526
Hsp90
Nfat (family)
caspase
HTRA2
−2.401
C/ebp
ADH1B
12.856
ALDH3A1
3.328
BLVRA
2.662
Nucleus
Ap1
Cyclin E
Cyclin A
NUMA1
−3.257
DACH1
−3.026

Figure S4:

IPA network 11: cell cycle, visual system development and function, hair and skin development and function

Functional annotation networks from IPA that show relationships among the genes that in IPA's relational database are related to cell cycle, visual system development and function, hair and skin development and function.

Solid lines indicated a direct interaction between proteins, while dotted lines indicate an indirect association between two proteins.

Proteins upregulated in the BALF dataset are shaded in red and proteins downregulated in the BALF dataset are shaded in green. The darker shading to lighter shading corresponds to decreasing expression intensity.

IPA=Ingenuity Pathway Analysis, BALF= Bronchoalveolar Lavage Fluid

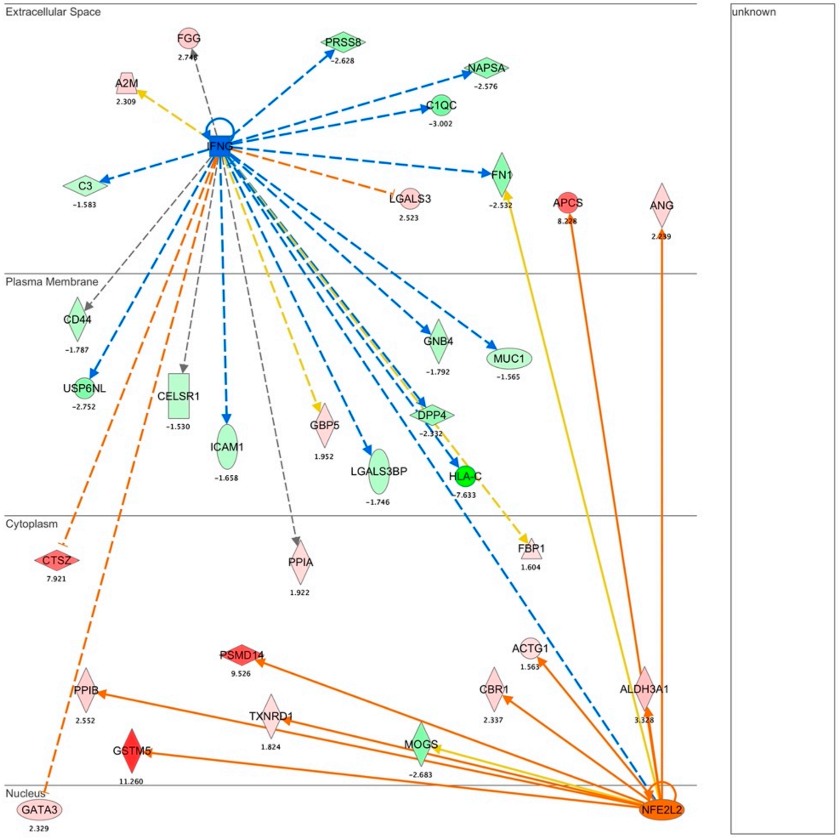

Extracellular Space
unknown
FGG
PRSS8
-2.628
NAPSA
-2.576
A2M
2.309
C1QC
-3.002
IFNG
C3
-1.583
LGALS3
2.523
FN1
-2.532
APCS
ANG
Plasma Membrane
CD44
-1.787
GNB4
-1.792
MUC1
-1.565
USP6NL
-2.752
CELSR1
-1.530
GBP5
1.952
DPP4
ICAM1
-1.658
LGALS3BP
-1.746
HLA-C
Cytoplasm
FBP1
1.604
CTSZ
7.921
PPIA
1.922
ACTG1
PSMD14
9.526
CBR1
2.337
ALDH3A1
PPIB
2.552
TXNRD1
1.824
MOGS
-2.683
GSTM5
11.260
Nucleus
GATA3
2.329
NFE2L2

Figure S5:

IPA network putative upstream regulators

Putative upstream regulators of the proteins that were significantly differentially expressed between the cohorts were associated with a predicted downregulation and upregulation of interferon gamma and nuclear factor erythroid 2-related 2 (NRF2) respectively.

Solid lines indicated a direct interaction between proteins, while dotted lines indicate an indirect association between two proteins. Proteins upregulated in the BALF dataset and are predicted are shaded in red and proteins downregulated in the BALF dataset are shaded in green. Interferon gamma is shaded in blue to denote a putative downregulation of the protein while NRF2 is shaded in orange to denote a putative upregulation of the protein based on the IPA relational database. The darker shading to lighter shading corresponds to decreasing expression intensity. The lines colored with orange shading correspond to interactions of the upstream regulator leading to increased protein synthesis of the downstream protein. The lines colored with blue shading correspond to interactions of the upstream regulator leading to decreased protein synthesis of the downstream protein. Lines colored with yellow shading indicate a downstream protein expression level that is discordant with the putative interaction from the upstream protein.

IPA=Ingenuity Pathway Analysis, BALF= Bronchoalveolar Lavage Fluid]

1. Berenson CS, Garlipp MA, Grove LJ, Maloney J, Sethi S. Impaired phagocytosis of nontypeable Haemophilus influenzae by human alveolar macrophages in chronic obstructive pulmonary disease. *J Infect Dis.* 2006;194(10):1375-1384.
2. Davis AP, Grondin CJ, Johnson RJ, et al. Comparative toxicogenomics database (CTD): update 2021. *Nucleic acids research.* 2021;49(D1):D1138-D1143.
3. Harju T, Mazur W, Merikallio H, Soini Y, Kinnula VL. Glutathione-S-transferases in lung and sputum specimens, effects of smoking and COPD severity. *Respiratory Research.* 2008;9:80.
4. Repapi E, Sayers I, Wain LV, et al. Genome-wide association study identifies five loci associated with lung function. *Nature Genetics.* 2010;42(1):36-44.
5. Soler Artigas M, Wain LV, Repapi E, et al. Effect of five genetic variants associated with lung function on the risk of chronic obstructive lung disease, and their joint effects on lung function. *American Journal of Respiratory & Critical Care Medicine.* 2011;184(7):786-795.
6. Mallia P, Footitt J, Sotero R, et al. Rhinovirus infection induces degradation of antimicrobial peptides and secondary bacterial infection in chronic obstructive pulmonary disease. *American Journal of Respiratory & Critical Care Medicine.* 2012;186(11):1117-1124.
7. Mantovani A. Pentraxin-3 in COPD: innocent bystander or amplifier? *Eur. Resp. J.* 2012;39(4):795-796.
8. Van Pottelberge GR, Bracke KR, Pauwels NS, Vermassen FE, Joos GF, Brusselle GG. COPD is associated with reduced pulmonary interstitial expression of pentraxin-3. *Eur. Resp. J.* 2012;39(4):830-838.
9. Di Stefano A, Caramori G, Gnemmi I, et al. Association of increased CCL5 and CXCL7 chemokine expression with neutrophil activation in severe stable COPD. *Thorax.* 2009;64(11):968-975.
10. Maltais F, Sullivan MJ, LeBlanc P, et al. Altered expression of myosin heavy chain in the vastus lateralis muscle in patients with COPD. *Eur. Resp. J.* 1999;13(4):850-854.
11. Nguyen T, Shrager J, Kaiser L, et al. Developmental myosin heavy chains in the adult human diaphragm: coexpression patterns and effect of COPD. *J. Appl. Physiol.* 2000;88(4):1446-1456.
12. Satta A, Migliori GB, Spanevello A, et al. Fibre types in skeletal muscles of chronic obstructive pulmonary disease patients related to respiratory function and exercise tolerance. *Eur. Resp. J.* 1997;10(12):2853-2860.
13. Verrills NM, Irwin JA, He XY, et al. Identification of novel diagnostic biomarkers for asthma and chronic obstructive pulmonary disease. *Am J Respir Crit Care Med.* 2011;183(12):1633-1643.
14. Engstrom G, Segelstorm N, Ekberg-Aronsson M, Nilsson PM, Lindgarde F, Lofdahl CG. Plasma markers of inflammation and incidence of hospitalisations for COPD: results from a population-based cohort study. *Thorax.* 2009;64(3):211-215.
15. van Dijk WD, Akkermans R, Heijdra Y, et al. The acute effect of cigarette smoking on the high-sensitivity CRP and fibrinogen

biomarkers in chronic obstructive pulmonary disease patients. *Biomarkers in Medicine.* 2013;7(2):211-219.

16. Duvoix A, Dickens J, Haq I, et al. Blood fibrinogen as a biomarker of chronic obstructive pulmonary disease. *Thorax.* 2013;68(7):670-676.
17. Mador MJ, Sethi S. Systemic inflammation in predicting COPD exacerbations. *JAMA.* 2013;309(22):2390-2391.
18. Thomsen M, Ingebrigtsen TS, Marott JL, et al. Inflammatory biomarkers and exacerbations in chronic obstructive pulmonary disease. *JAMA.* 2013;309(22):2353-2361.
19. Celli BR, Locantore N, Yates J, et al. Inflammatory biomarkers improve clinical prediction of mortality in chronic obstructive pulmonary disease. *Am J Respir Crit Care Med.* 2012;185(10):1065-1072.
20. Thomsen M, Dahl M, Lange P, Vestbo J, Nordestgaard BG. Inflammatory biomarkers and comorbidities in chronic obstructive pulmonary disease. *Am J Respir Crit Care Med.* 2012;186(10):982-988.
21. Gorowiec MR, Borthwick LA, Parker SM, Kirby JA, Saretzki GC, Fisher AJ. Free radical generation induces epithelial-to-mesenchymal transition in lung epithelium via a TGF-1-dependent mechanism. *Free Radical Biology & Medicine.* 2012;52(6):1024-1032.
22. Milara J, Peiro T, Serrano A, Cortijo J. Epithelial to mesenchymal transition is increased in patients with COPD and induced by cigarette smoke. *Thorax.* 2013;68(5):410-420.
23. Sohal SS, Reid D, Soltani A, et al. Reticular basement membrane fragmentation and potential epithelial mesenchymal transition is exaggerated in the airways of smokers with chronic obstructive pulmonary disease. *Respirology.* 2010;15(6):930-938.
24. Wang Q, Wang Y, Zhang Y, Zhang Y, Xiao W. The role of uPAR in epithelial-mesenchymal transition in small airway epithelium of patients with chronic obstructive pulmonary disease. *Respiratory Research.* 2013;14:67.
25. Zou W, Zou Y, Zhao Z, Li B, Ran P. Nicotine-induced epithelial-mesenchymal transition via Wnt/-catenin signaling in human airway epithelial cells. *American Journal of Physiology - Lung Cellular & Molecular Physiology.* 2013;304(4):L199-209.
26. Mukaro VR, Bylund J, Hodge G, et al. Lectins offer new perspectives in the development of macrophage-targeted therapies for COPD/emphysema. *PLoS ONE [Electronic Resource].* 2013;8(2):e56147.
27. Pilette C, Colinet B, Kiss R, et al. Increased galectin-3 expression and intra-epithelial neutrophils in small airways in severe COPD. *Eur. Resp. J.* 2007;29(5):914-922.
28. Brissenden JE, Cox DW. alpha 2-Macroglobulin in patients with obstructive lung disease, with and without alpha 1-antitrypsin deficiency. *Clinica Chimica Acta.* 1983;128(2-3):241-248.
29. Kilroe-Smith TA, Dowdeswell RJ, Gaillard MC. Elastase binding capacity of alpha 2-macroglobulin in plasma of patients with asthma or chronic obstructive pulmonary disease, without alpha 1-protease inhibitor deficiency. *Clinica Chimica Acta.* 1989;185(1):81-90.

30. Mocchegiani E, Giacconi R, Costarelli L. Metalloproteases/anti-metalloproteases imbalance in chronic obstructive pulmonary disease: genetic factors and treatment implications. *Current Opinion in Pulmonary Medicine.* 2011;17 Suppl 1:S11-19.
31. Poller W, Barth J, Voss B. Detection of an alteration of the alpha 2-macroglobulin gene in a patient with chronic lung disease and serum alpha 2-macroglobulin deficiency. *Human Genetics.* 1989;83(1):93-96.
32. Ekeowa UI, Gooptu B, Belorgey D, et al. alpha1-Antitrypsin deficiency, chronic obstructive pulmonary disease and the serpinopathies. *Clinical Science.* 2009;116(12):837-850.
33. Lomas DA. Parker B. Francis lectureship. Antitrypsin deficiency, the serpinopathies, and chronic obstructive pulmonary disease. *Proceedings of the American Thoracic Society.* 2006;3(6):499-501.
34. Kristan SS, Marc MM, Kern I, et al. Airway angiogenesis in stable and exacerbated chronic obstructive pulmonary disease. *Scandinavian Journal of Immunology.* 2012;75(1):109-114.
35. Nicholas BL, Skipp P, Barton S, et al. Identification of lipocalin and apolipoprotein A1 as biomarkers of chronic obstructive pulmonary disease. *American Journal of Respiratory & Critical Care Medicine.* 2010;181(10):1049-1060.
36. Yoshida S, Minematsu N, Chubachi S, et al. Annexin V decreases PS-mediated macrophage efferocytosis and deteriorates elastase-induced pulmonary emphysema in mice. *American Journal of Physiology - Lung Cellular & Molecular Physiology.* 2012;303(10):L852-860.
37. Savarimuthu Francis SM, Larsen JE, Pavey SJ, et al. Genes and gene ontologies common to airflow obstruction and emphysema in the lungs of patients with COPD. *PLoS ONE [Electronic Resource].* 2011;6(3):e17442.
38. Annoni R, Lancas T, Yukimatsu Tanigawa R, et al. Extracellular matrix composition in COPD. *Eur. Resp. J.* 2012;40(6):1362-1373.
39. Hu R, Ouyang Q, Dai A, Tan S, Xiao Z, Tang C. Heat shock protein 27 and cyclophilin A associate with the pathogenesis of COPD. *Respirology.* 2011;16(6):983-993.
40. Gosker HR, Langen RC, Bracke KR, et al. Extrapulmonary manifestations of chronic obstructive pulmonary disease in a mouse model of chronic cigarette smoke exposure. *American Journal of Respiratory Cell & Molecular Biology.* 2009;40(6):710-716.
41. Gosker HR, Zeegers MP, Wouters EF, Schols AM. Muscle fibre type shifting in the vastus lateralis of patients with COPD is associated with disease severity: a systematic review and meta-analysis. *Thorax.* 2007;62(11):944-949.
42. Jackson AS, Shrikrishna D, Kelly JL, et al. Vitamin D and skeletal muscle strength and endurance in COPD.[Erratum appears in Eur Respir J. 2013 Apr;41(4):998 Note: Kemp, Samuel V [added]]. *Eur. Resp. J.* 2013;41(2):309-316.
43. Levine S, Kaiser L, Leferovich J, Tikunov B. Cellular adaptations in the diaphragm in chronic obstructive pulmonary disease. *New England Journal of Medicine.* 1997;337(25):1799-1806.
44. Lewis MI, Fournier M, Storer TW, et al. Skeletal muscle adaptations to testosterone and resistance training in men with COPD. *J. Appl. Physiol.* 2007;103(4):1299-1310.

45. Ottenheijm CA, Heunks LM, Sieck GC, et al. Diaphragm dysfunction in chronic obstructive pulmonary disease. *American Journal of Respiratory & Critical Care Medicine.* 2005;172(2):200-205.
46. Basic VT, Tadele E, Elmabsout AA, et al. Exposure to cigarette smoke induces overexpression of von Hippel-Lindau tumor suppressor in mouse skeletal muscle. *American Journal of Physiology - Lung Cellular & Molecular Physiology.* 2012;303(6):L519-527.
47. Holownia A, Mroz RM, Kielek A, Chyczewska E, Braszko JJ. Nuclear HSP90 and HSP70 in COPD patients treated with formoterol or formoterol and corticosteroids. *European Journal of Medical Research.* 2009;14 Suppl 4:104-107.
48. Matokanovic M, Rumora L, Popovic-Grle S, Cepelak I, Culic O, Barisic K. Association of hsp70-2 (+1267A/G), hsp70-hom (+2437T/C), HMOX-1 (number of GT repeats) and TNF-alpha (+489G/A) polymorphisms with COPD in Croatian population. *Clinical Biochemistry.* 2012;45(10-11):770-774.
49. Xie J, Zhao J, Xiao C, Xu Y, Yang S, Ni W. Reduced heat shock protein 70 in airway smooth muscle in patients with chronic obstructive pulmonary disease. *Exp. Lung Res.* 2010;36(4):219-226.
50. Andresen E, Lange C, Strodthoff D, et al. S100A7/psoriasin expression in the human lung: unchanged in patients with COPD, but upregulated upon positive S. aureus detection. *BMC Pulmonary Medicine.* 2011;11:10.
51. Cockayne DA, Cheng DT, Waschki B, et al. Systemic biomarkers of neutrophilic inflammation, tissue injury and repair in COPD patients with differing levels of disease severity. *PLoS ONE [Electronic Resource].* 2012;7(6):e38629.
52. Gray RD, MacGregor G, Noble D, et al. Sputum proteomics in inflammatory and suppurative respiratory diseases. *American Journal of Respiratory & Critical Care Medicine.* 2008;178(5):444-452.
53. Qiu W, Cho MH, Riley JH, et al. Genetics of sputum gene expression in chronic obstructive pulmonary disease. *PLoS ONE [Electronic Resource].* 2011;6(9):e24395.
54. Guo X, Lin HM, Lin Z, et al. Surfactant protein gene A, B, and D marker alleles in chronic obstructive pulmonary disease of a Mexican population. *Eur. Resp. J.* 2001;18(3):482-490.
55. Ilumets H, Mazur W, Toljamo T, et al. Ageing and smoking contribute to plasma surfactant proteins and protease imbalance with correlations to airway obstruction. *BMC Pulmonary Medicine.* 2011;11:19.
56. Ishikawa N, Hattori N, Tanaka S, et al. Levels of surfactant proteins A and D and KL-6 are elevated in the induced sputum of chronic obstructive pulmonary disease patients: a sequential sputum analysis. *Respiration.* 2011;82(1):10-18.
57. Larsson P, Mirgorodskaya E, Samuelsson L, et al. Surfactant protein A and albumin in particles in exhaled air. *Respiratory Medicine.* 2012;106(2):197-204.
58. Ohlmeier S, Vuolanto M, Toljamo T, et al. Proteomics of human lung tissue identifies surfactant protein A as a marker of chronic obstructive pulmonary disease. *Journal of Proteome Research.* 2008;7(12):5125-5132.
59. van Diemen CC, Postma DS, Aulchenko YS, et al. Novel strategy to identify genetic risk factors for COPD severity: a genetic

isolate. *Eur. Resp. J.* 2010;35(4):768-775.

60. Vlachaki EM, Koutsopoulos AV, Tzanakis N, et al. Altered surfactant protein-A expression in type II pneumocytes in COPD. *Chest.* 2010;137(1):37-45.
61. Larocca NE, Moreno D, Garmendia JV, De Sanctis JB. Inhibitors of phosphoinositol 3 kinase and NFkB for the treatment of chronic obstructive pulmonary disease. *Recent Patents on Inflammation & Allergy Drug Discovery.* 2011;5(3):178-183.
62. Marwick JA, Caramori G, Casolari P, et al. A role for phosphoinositol 3-kinase delta in the impairment of glucocorticoid responsiveness in patients with chronic obstructive pulmonary disease. *Journal of Allergy & Clinical Immunology.* 2010;125(5):1146-1153.
63. To Y, Ito K, Kizawa Y, et al. Targeting phosphoinositide-3-kinase-delta with theophylline reverses corticosteroid insensitivity in chronic obstructive pulmonary disease. *American Journal of Respiratory & Critical Care Medicine.* 2010;182(7):897-904.
64. Chung S, Sundar IK, Hwang JW, et al. NF-kB inducing kinase, NIK mediates cigarette smoke/TNF-induced histone acetylation and inflammation through differential activation of IKKs. *PLoS ONE [Electronic Resource].* 2011;6(8):e23488.
65. Gagliardo R, Chanez P, Profita M, et al. IkB kinase-driven nuclear factor-kB activation in patients with asthma and chronic obstructive pulmonary disease. *Journal of Allergy & Clinical Immunology.* 2011;128(3):635-645.e631-632.
66. Yao H, Chung S, Hwang JW, et al. SIRT1 protects against emphysema via FOXO3-mediated reduction of premature senescence in mice. *Journal of Clinical Investigation.* 2012;122(6):2032-2045.
67. Harvey CJ, Thimmulappa RK, Sethi S, et al. Targeting Nrf2 signaling improves bacterial clearance by alveolar macrophages in patients with COPD and in a mouse model. *Science Translational Medicine.* 2011;3(78):78ra32.
68. Thomsen M, Nordestgaard BG, Kobzik L, Dahl M. Genetic variation in the scavenger receptor MARCO and its association with chronic obstructive pulmonary disease and lung infection in 10,604 individuals. *Respiration.* 2013;85(2):144-153.
69. Baarsma HA, Spanjer AI, Haitsma G, et al. Activation of WNT/-catenin signaling in pulmonary fibroblasts by TGF-1 is increased in chronic obstructive pulmonary disease. *PLoS ONE [Electronic Resource].* 2011;6(9):e25450.
70. Krimmer DI, Burgess JK, Wooi TK, Black JL, Oliver BG. Matrix proteins from smoke-exposed fibroblasts are pro-proliferative. *American Journal of Respiratory Cell & Molecular Biology.* 2012;46(1):34-39.
71. Man SF, Xing L, Connett JE, et al. Circulating fibronectin to C-reactive protein ratio and mortality: a biomarker in COPD? *Eur. Resp. J.* 2008;32(6):1451-1457.
72. Michalski J, Kanaji N, Liu X, et al. Attenuation of inhibitory prostaglandin E2 signaling in human lung fibroblasts is mediated by phosphodiesterase 4. *American Journal of Respiratory Cell & Molecular Biology.* 2012;47(6):729-737.
73. Tomic R, Lassiter CC, Ritzenthaler JD, Rivera HN, Roman J. Anti-tissue remodeling effects of corticosteroids: fluticasone propionate inhibits fibronectin expression in fibroblasts. *Chest.* 2005;127(1):257-265.
74. Bowler RP. Surfactant protein D as a biomarker for chronic obstructive pulmonary disease. *Copd: Journal of Chronic*

Obstructive Pulmonary Disease. 2012;9(6):651-653.
75. Foreman MG, Kong X, DeMeo DL, et al. Polymorphisms in surfactant protein-D are associated with chronic obstructive pulmonary disease. *American Journal of Respiratory Cell & Molecular Biology.* 2011;44(3):316-322.
76. Kim DK, Cho MH, Hersh CP, et al. Genome-wide association analysis of blood biomarkers in chronic obstructive pulmonary disease. *American Journal of Respiratory & Critical Care Medicine.* 2012;186(12):1238-1247.
77. Lomas DA, Silverman EK, Edwards LD, et al. Serum surfactant protein D is steroid sensitive and associated with exacerbations of COPD. *Eur. Resp. J.* 2009;34(1):95-102.
78. More JM, Voelker DR, Silveira LJ, Edwards MG, Chan ED, Bowler RP. Smoking reduces surfactant protein D and phospholipids in patients with and without chronic obstructive pulmonary disease. *BMC Pulmonary Medicine.* 2010;10:53.
79. Shakoori TA, Sin DD, Bokhari SN, Ghafoor F, Shakoori AR. SP-D polymorphisms and the risk of COPD. *Disease Markers.* 2012;33(2):91-100.
80. Kaparianos A, Argyropoulou E. Local renin-angiotensin II systems, angiotensin-converting enzyme and its homologue ACE2: their potential role in the pathogenesis of chronic obstructive pulmonary diseases, pulmonary hypertension and acute respiratory distress syndrome. *Current Medicinal Chemistry.*18(23):3506-3515.
81. Somborac-Bacura A, Buljevic S, Rumora L, et al. Decreased soluble dipeptidyl peptidase IV activity as a potential serum biomarker for COPD. *Clinical Biochemistry.* 2012;45(15):1245-1250.
82. Dahlen I, Janson C, Bjornsson E, Stalenheim G, Peterson CG, Venge P. Changes in inflammatory markers following treatment of acute exacerbations of obstructive pulmonary disease. *Respiratory Medicine.* 2001;95(11):891-897.
83. Hodge S, Hodge G, Ahern J, Jersmann H, Holmes M, Reynolds PN. Smoking alters alveolar macrophage recognition and phagocytic ability: implications in chronic obstructive pulmonary disease. *American Journal of Respiratory Cell & Molecular Biology.* 2007;37(6):748-755.
84. Klagas I, Goulet S, Karakiulakis G, et al. Decreased hyaluronan in airway smooth muscle cells from patients with asthma and COPD. *Eur. Resp. J.* 2009;34(3):616-628.
85. Noguera A, Gomez C, Faner R, et al. An investigation of the resolution of inflammation (catabasis) in COPD. *Respiratory Research.* 2012;13:101.
86. Pons AR, Noguera A, Blanquer D, Sauleda J, Pons J, Agusti AG. Phenotypic characterisation of alveolar macrophages and peripheral blood monocytes in COPD. *Eur. Resp. J.* 2005;25(4):647-652.
87. Hollander C, Sitkauskiene B, Sakalauskas R, Westin U, Janciauskiene SM. Serum and bronchial lavage fluid concentrations of IL-8, SLPI, sCD14 and sICAM-1 in patients with COPD and asthma. *Respiratory Medicine.* 2007;101(9):1947-1953.
88. Keicho N, Elliott WM, Hogg JC, Hayashi S. Adenovirus E1A gene dysregulates ICAM-1 expression in transformed pulmonary epithelial cells. *American Journal of Respiratory Cell & Molecular Biology.* 1997;16(1):23-30.

89. Kim S, Nadel JA. Fibrinogen binding to ICAM-1 promotes EGFR-dependent mucin production in human airway epithelial cells. *American Journal of Physiology - Lung Cellular & Molecular Physiology.* 2009;297(1):L174-183.
90. Lopez-Campos JL, Calero C, Arellano-Orden E, et al. Increased levels of soluble ICAM-1 in chronic obstructive pulmonary disease and resistant smokers are related to active smoking. *Biomarkers in Medicine.* 2012;6(6):805-811.
91. Riise GC, Larsson S, Lofdahl CG, Andersson BA. Circulating cell adhesion molecules in bronchial lavage and serum in COPD patients with chronic bronchitis. *Eur. Resp. J.* 1994;7(9):1673-1677.
92. Rusznak C, Mills PR, Devalia JL, Sapsford RJ, Davies RJ, Lozewicz S. Effect of cigarette smoke on the permeability and IL-1beta and sICAM-1 release from cultured human bronchial epithelial cells of never-smokers, smokers, and patients with chronic obstructive pulmonary disease. *American Journal of Respiratory Cell & Molecular Biology.* 2000;23(4):530-536.
93. Sajjan US, Jia Y, Newcomb DC, et al. H. influenzae potentiates airway epithelial cell responses to rhinovirus by increasing ICAM-1 and TLR3 expression. *FASEB Journal.* 2006;20(12):2121-2123.
94. Schneider D, Ganesan S, Comstock AT, et al. Increased cytokine response of rhinovirus-infected airway epithelial cells in chronic obstructive pulmonary disease. *American Journal of Respiratory & Critical Care Medicine.* 2010;182(3):332-340.
95. Yamaya M, Nishimura H, Hatachi Y, et al. Inhibitory effects of tiotropium on rhinovirus infection in human airway epithelial cells. *Eur. Resp. J.* 2012;40(1):122-132.
96. Zandvoort A, van der Geld YM, Jonker MR, et al. High ICAM-1 gene expression in pulmonary fibroblasts of COPD patients: a reflection of an enhanced immunological function. *Eur. Resp. J.* 2006;28(1):113-122.
97. Letuve S, Kozhich A, Arouche N, et al. YKL-40 is elevated in patients with chronic obstructive pulmonary disease and activates alveolar macrophages. *Journal of Immunology.* 2008;181(7):5167-5173.
98. Otsuka K, Matsumoto H, Niimi A, et al. Sputum YKL-40 levels and pathophysiology of asthma and chronic obstructive pulmonary disease. *Respiration.* 2012;83(6):507-519.
99. Sakazaki Y, Hoshino T, Takei S, et al. Overexpression of chitinase 3-like 1/YKL-40 in lung-specific IL-18-transgenic mice, smokers and COPD. *PLoS ONE [Electronic Resource].* 2011;6(9):e24177.
100. Kosmas EN, Zorpidou D, Vassilareas V, Roussou T, Michaelides S. Decreased C4 complement component serum levels correlate with the degree of emphysema in patients with chronic bronchitis. *Chest.* 1997;112(2):341-347.
101. Miller RD, Kueppers F, Offord KP. Serum concentrations of C3 and C4 of the complement system in patients with chronic obstructive pulmonary disease. *Journal of Laboratory & Clinical Medicine.* 1980;95(2):266-271.
102. Stockley RA, Mistry M, Bradwell AR, Burnett D. A study of plasma proteins in the sol phase of sputum from patients with chronic bronchitis. *Thorax.* 1979;34(6):777-782.
103. Barta P, Van Pelt C, Men T, Dickey BF, Lotan R, Moghaddam SJ. Enhancement of lung tumorigenesis in a Gprc5a Knockout mouse by chronic extrinsic airway inflammation. *Molecular cancer.* 2012;11:4.

104. Fujimoto J, Kadara H, Garcia MM, et al. G-protein coupled receptor family C, group 5, member A (GPRC5A) expression is decreased in the adjacent field and normal bronchial epithelia of patients with chronic obstructive pulmonary disease and non-small-cell lung cancer. *Journal of Thoracic Oncology: Official Publication of the International Association for the Study of Lung Cancer.* 2012;7(12):1747-1754.
105. Ishikawa N, Mazur W, Toljamo T, et al. Ageing and long-term smoking affects KL-6 levels in the lung, induced sputum and plasma. *BMC Pulmonary Medicine.* 2011;11:22.
106. Leikauf GD, Borchers MT, Prows DR, Simpson LG. Mucin apoprotein expression in COPD. *Chest.* 2002;121(5 Suppl):166S-182S.
107. Freeman CM, Curtis JL, Chensue SW. CC chemokine receptor 5 and CXC chemokine receptor 6 expression by lung CD8+ cells correlates with chronic obstructive pulmonary disease severity. *American Journal of Pathology.* 2007;171(3):767-776.
108. Su YW, Xu YJ, Liu XS. Quantitative differentiation of dendritic cells in lung tissues of smokers with and without chronic obstructive pulmonary disease. *Chinese Medical Journal.* 2010;123(12):1500-1504.
109. Tsoumakidou M, Koutsopoulos AV, Tzanakis N, et al. Decreased small airway and alveolar CD83+ dendritic cells in COPD. *Chest.* 2009;136(3):726-733.
110. Verhoeven GT, Hegmans JP, Mulder PG, Bogaard JM, Hoogsteden HC, Prins JB. Effects of fluticasone propionate in COPD patients with bronchial hyperresponsiveness. *Thorax.* 2002;57(8):694-700.
111. Shapiro SD. Proteolysis in the lung. *The European respiratory journal. Supplement.* 2003;44(44 suppl):30s-32s.
112. Annoni R, Lancas T, Yukimatsu Tanigawa R, et al. Extracellular matrix composition in COPD. *Eur Respir J.* 2012;40(6):1362-1373.
113. Riise GC, Larsson S, Lofdahl CG, Andersson BA. Circulating cell adhesion molecules in bronchial lavage and serum in COPD patients with chronic bronchitis. *Eur Respir J.* 1994;7(9):1673-1677.
114. Yang M, Kohler M, Heyder T, et al. Proteomic profiling of lung immune cells reveals dysregulation of phagocytotic pathways in female-dominated molecular COPD phenotype. *Respir Res.* 2018;19(1):39.
115. Sethi S. Bacterial infection and the pathogenesis of COPD. *Chest.* 2000;117(5 Suppl 1):286S-291S.
116. Stockley RA. Neutrophils and the pathogenesis of COPD. *Chest.* 2002;121(5 Suppl):151S-155S.
117. Tu C, Mammen MJ, Li J, et al. Large-scale, ion-current-based proteomics investigation of bronchoalveolar lavage fluid in chronic obstructive pulmonary disease patients. *J Proteome Res.* 2014;13(2):627-639.
